# Trajectory of suicide among Indian children and adolescents: a pooled analysis of national data from 1995 to 2021

**DOI:** 10.1186/s13034-024-00818-9

**Published:** 2024-09-30

**Authors:** Susangita Jena, Prafulla Kumar Swain, Rachel Elizabeth Senapati, Subhendu Kumar Acharya

**Affiliations:** 1 ICMR-Regional Medical Research Centre, NALCO Nagar, Chandrasekharpur, Bhubaneswar, 751023 Odisha India; 2 Department of Statistics, Utkal University, Bhubaneswar, Odisha India

**Keywords:** Children and adolescent suicide, Self-harm, Mental health illness, NCRB, India

## Abstract

**Background:**

Suicide is a major public health concern in India especially among children and adolescents. The yearly national statistics show a concerning trend of rising suicide deaths in these age groups.

**Methods:**

The present study, taking 26 years of national data from the National Crime Record Bureau during 1995–2021, examined the trend, patterns, means, and modes of children/adolescent suicides in India. We also undertook a time series analysis by using ARIMA (0,2,1) model to forecast the expected suicide rate for the next one decade.

**Results:**

A rising trend of suicide rate among children and adolescents was observed in India over the last 26 years. The forecast indicates a continuance of rising suicide cases for the upcoming decade in India. A substantially different trend of suicide rate was observed among early and late adolescents indicating significantly high vulnerability of late adolescents. Among children /adolescents, the most common causes of suicide were family problems, academic failure, illness, and unemployment. Illness has emerged as one of the leading causes of suicide, with a significant rise over time. Poverty and unemployment were also found as the important contributors with a steadily increasing trend of suicide among children and adolescents facing these problems in recent years.

**Conclusion:**

The study provides important analysis and information on suicide among children/adolescents in India, by providing useful insights for parents, teachers, policymakers, healthcare practitioners, and stakeholders aiming to prevent and control children and adolescent suicide and boost mental health. The study also provides important leads on risk factors with a forecast of suicide trends for the next 10 years.

**Supplementary Information:**

The online version contains supplementary material available at 10.1186/s13034-024-00818-9.

## Introduction

Suicide among children and adolescents is a significant public health concern globally, with wide-ranging social, political, and emotional implications [1] A pooled analysis of the Global School-Based Survey (GSHS) across 90 countries revealed a significant prevalence of suicidal ideation among 397,299 adolescents [2]. The majority of suicides occur in low- and middle-income countries (LMICs) in the world [3]; India witnessed a constantly rising suicide rate over the past three decades with the highest number of incidences in the world [4].

**Fig. 1:**
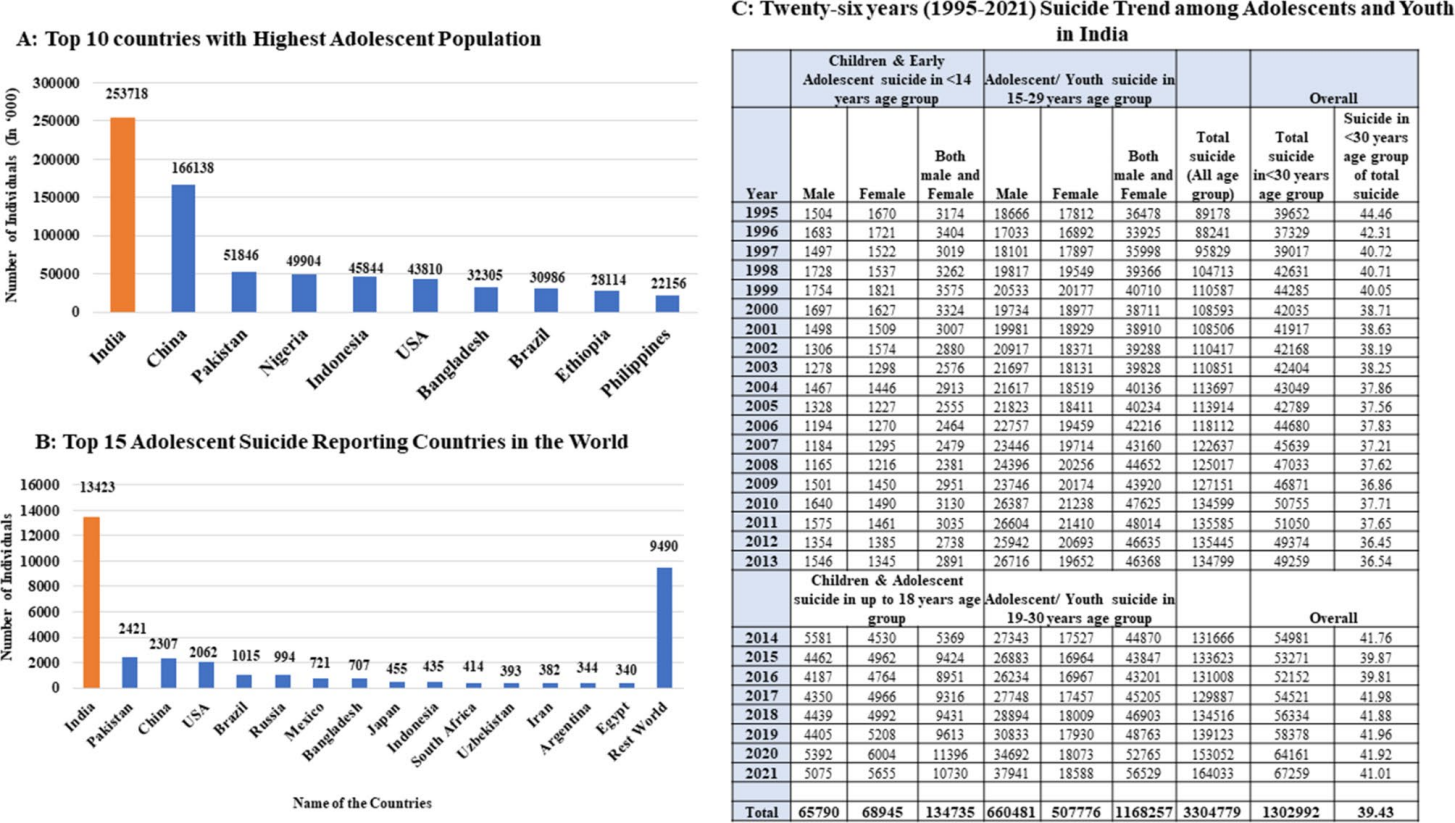
**A**, **B** and **C**): Top 10 Countries with Highest Adolescent Population (**A**), Top 15 adolescent suicide reporting countries in the world (**B**), and T 26 years (1995–2021) suicide trend among children, adolescents, and Youth in India (**C**).

The suicide scenario among Indian children, adolescents and youth (< 30 years old) is highly alarming as it is their top leading cause of death [5–7] (Fig. 1C). It is here noteworthy that India with 253 million children and adolescent age constitutes one-fifth and the largest proportion of the adolescent population in the world [5]. It has been observed that 40% of suicide deaths in men and 56% of suicide deaths in women occurred between 15 and 29 years of age [8]. A systematic review and meta-analysis revealed a high burden of psychiatric disorders among Indian adolescents in the community (6.5%) and school settings (23.3%) as well as urban (0.8–29.4%) and rural areas (1.60–5.84%) [9]. Recent reviews of risk factors of adolescent suicide scenarios in India revealed that the most frequently reported risk factors included; mental health problems, negative life issues, academic stressors, violence, economic distress, relationship factors, etc [10, 11].

At the same time, mental health in India is also neglected and inadequately understood across all age groups [12, 13]. Youths in India generally view suicide as morally unacceptable and heavily stigmatized [14]. Adolescents and their issues in Indian culture are not taken seriously as they are considered naive and exaggerated, for which they must be put to face life’s challenges, most importantly with the least support, to learn from adversities [15]. Furthermore, misconceptions surrounding mental health coupled with poor reporting, poor treatment-seeking practices, and non-adherence to treatment in case of psychiatric need/diseases are deep-rooted and widespread concerns [8].

As per our recent scoping review, there is a substantial gap in adolescent mental health research in India with a major vacuum of evidence around the status, major risk factors, and future risks of suicide at the national population level [10]. In this context, this study examines the detailed historical and recent trends and causes of children and adolescent suicide rates stratified by age and sex in India over the last 26-year period (from 1995 to 2021). Additionally, the paper also tries to understand the future trend of children and adolescent suicide by forecasting the suicide rate for the next 10 years by using time series data. The study based on previous reported findings and our primary experience aims to identify and contribute valuable insights to healthcare planning and suicide prevention programs.

## Methodology

### Study design and study settings

The study employed a retrospective cross-sectional analysis design utilizing data on reported adolescent suicides across India for a period of 26 years from 1995 to 2021. The data indicates that there are nearly 0.2 million reported suicides during this timeframe.

### Study participants

National Crime Record Bureau (NCRB) in its report “Accidental Deaths and Suicides in India (ADSI)” stratified and reported the suicide data on children up to 14 years of both male and females till the year 2023; from the year 2014 onwards, the age group for children and adolescent was extended up to 18 years. So, our analysis included the data accordingly.

### Data source and data extraction

We collected the data from the NCRB, India, the primary body for gathering information on suicide incidences across India. National Crime Record Bureau (NCRB) is part of the Ministry of Home Affairs, Government of India and it releases yearly time series data on accidental deaths and suicides from the year 1969 onwards. All the states and union territories were covered during the study. For this study, we gathered data on children’s suicide for a period of 26 years (1995–2021) from NCRB annual reports on Accidental Deaths and Suicides in India (ADSI), freely available at https://ncrb.gov.in/en/adsi-reports-of-previous-years [16]. The data of the period 1995–2013 reported the year-wise suicide among children below 14 years of age with information on both causes and means of suicide. Year 2014 onwards, the NCRB reported suicide among children/adolescents up to 18 years old. However, they dropped the reporting of means and only reported the causes of suicide. So, we have analysed the data in two sets as 1995–2013 and 2014–2021 where required. We have also added a population figure at the time when the data was extracted for the analysis (Additional File 2).

Data have been extracted from NCRB reports, available as PDF documents, by two researchers (1st and 3rd authors). The year-wise suicide data was entered into a Microsoft Excel 2019 spreadsheet. The data extraction, validation, and entry were alternatively checked by two researchers (1st and 3rd authors) and were finally cross-checked by the corresponding author for consistency and correctness.

### Ethical approval statement

This research paper does not require ethical approval; it involves a systematic analysis and presentation of available secondary data resources.

### Statistical methods

The present study aimed to understand the patterns and trends of suicide among adolescents in India. We also intended to study the major risk factors in this process. In this context, we used descriptive analysis of the data to reach conclusions. We used various graphs to explain the patterns and trends of suicide in India; we used the adolescent population data available from UNICEF for calculating the country-wise adolescent population while the number of suicide was calculated by using the adolescent suicide rate and adolescent population of each country available from the same source [17, 18]. By using the data available at NCRB, Government of India, we prepared the 26 years (1995–2021) suicide trend among adolescents and youth in India. The heatmap method was used to highlight the trends and patterns for various modes and means of children/adolescent suicide (Figs. 4 and 5). The primary purpose of a heat map is to quickly and intuitively highlight areas of high or low concentration within the dataset to indicate the risks. Based on our analysis of the 26 years of data, we identified the top leading factors causing suicide which we illustrated with line graphs.

We calculated yearly crude suicide rates per 100,000 populations with 95% confidence intervals using projected mid-year population estimates. Also, the crude suicide rate per 100,000 population and trends were described using joint point regression analysis to determine the major deviation in trend during the 26 years (1995–20221), utilizing the Joint Point Regression Program, Version 4.5.0.1 (Surveillance Research Program, National Cancer Institute, USA) [19].

Moreover, we undertook a time series analysis and forecasting of suicide trends (for the next 10 years) based on available data. Time series forecasting is a process of evaluating previous observations of a time series, which is a series of data points collected over time, in order to construct a model that accurately describes its underlying structure. The aim is to use this model to forecast expected future trends of suicide risks among Indian adolescents. One of the most popular and frequently used stochastic time series models is the Auto-Regressive Integrated Moving Average (ARIMA) model [20] which we used for the present analysis and forecasting purpose. The ARIMA model has been successfully applied in the field of health as well as in different fields in the past due to its simple structure, fast applicability, and ability to explain the data set. The ARIMA model combines these three components to create a powerful forecasting tool. It considers past suicide rates (AR), adjusts for potential trends (I), and accounts for random fluctuations (MA) to provide a more accurate forecast of future suicide risk.

The statistical analysis and forecasting were carried out using various software tools, including Microsoft Excel 2019, and R software version 3.6.2.

## Results

The proportion of India’s adolescent population to that of the world, as per the recent data, stands highest at 253 million among all the countries followed by China at 168 million (Fig. 1A). The number of suicides among Indian adolescents stands the highest (10,730) than the second highest reporting country, Pakistan (2421) followed by China (Fig. 1B). As per the last 26 years of NCRB reports, 1,34,735 reported children and adolescents died by committing suicide in the country. By adjusting the data discrepancy, it can be observed that 1.3 million deaths among children and youth combined took place due to suicide during the same period. This number is about 40% of the approximately 3.3 million total suicides (including all age groups) in the country that happened in India during these 26 years (Fig. 1C).

1. Trends of children and adolescent suicide in India (1995–2021)

**Fig. 2:**
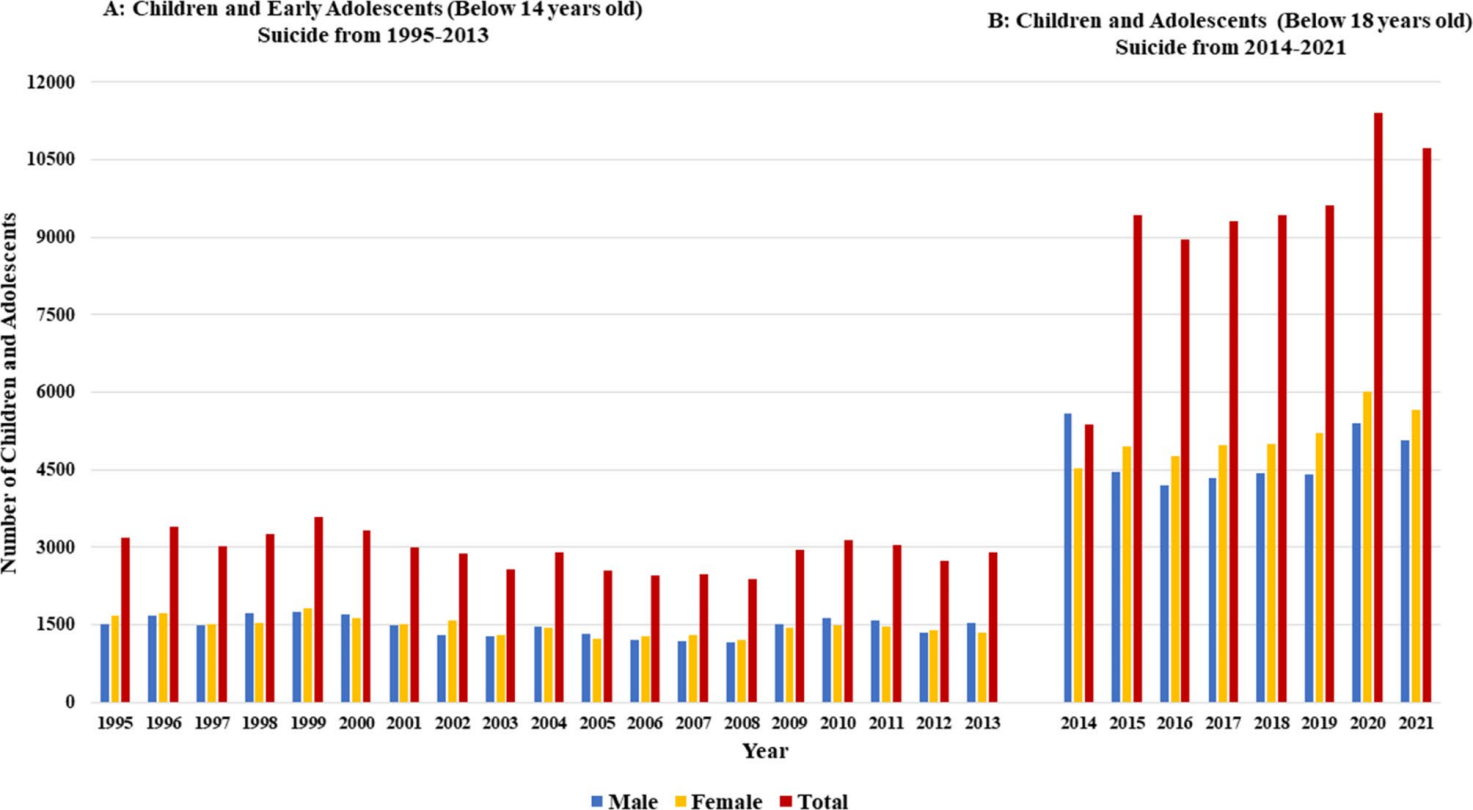
Historical and recent trends in children and adolescent suicide rate in India

Figure 2 illustrates the trends in children and adolescent suicide rates in India from 1995 to 2021. Where Fig. 2A describes the Trend in children and early adolescent suicide in India from 1995 to 2013, and Fig. 2B describes trends in Children and adolescents suicide in India from 2014 to 2021. The Bar graphs highlight a significant increase in the suicide rate among these vulnerable populations over 26 years. In Fig. 2A, the data on children and early adolescents from 1995 to 2013 shows a fluctuating suicide rate between males and females each year. However, Fig. 2B reveals a shift in the trend after 2014, indicating higher suicide rates among female children and adolescents compared to males. The latest data in 2021 shows that approximately 5075 males and 5655 females in India in this age group died by suicide.

Table 1 represents the data on yearly crude suicide rates per 100,000 population from 1995 to 2021 revealing significant fluctuation over the period. In the late 1990s, the rates were relatively similar, with a slight peak in 1999 at 7.53 [95% CI: 7.29, 7.78] and a dip in 1997 at 6.52 [95% CI: 6.28, 6.75]. A notable decline was observed from 2001 to 2008, with the lowest rate recorded in 2008 at 4.72 [95% CI: 4.53,4.91]. However, starting from 2009, there is a clear upward trend, culminating in a dramatic increase from 2014 onwards. The most substantial rise occurred in 2015, with a rate of 18.67 [95% CI: 18.29, 19.04], which continued to escalate, peaking in 2020 at 23.8 [95% CI: 22.6, 23.50]. A slight decrease was seen in 2021 with a rate of 21.9 [95% CI: 21.49, 22.31]. this trend indicates a worrying increase in suicide rates over the last decade.

**Table 1:** Year wise crude suicide rates per 100,000 populations with 95% confidence intervals [95% CI]

Year	Crude suicide rate	Lower confidential interval	Upper confidential interval
1995	7.03	6.79	7.27
1996	7.44	7.19	7.69
1997	6.52	6.28	6.75
1998	6.95	6.72	7.19
1999	7.53	7.29	7.78
2000	6.93	6.70	7.17
2001	6.21	5.99	6.43
2002	5.89	5.68	6.10
2003	5.22	5.02	5.43
2004	5.87	5.66	6.08
2005	5.12	4.92	5.32
2006	4.92	4.72	5.11
2007	4.93	4.74	5.12
2008	4.72	4.53	4.91
2009	5.84	5.63	6.05
2010	6.19	5.97	6.40
2011	5.99	5.78	6.20
2012	5.40	5.20	5.60
2013	5.70	5.50	5.91
2014	10.61	10.32	10.89
2015	18.67	18.29	19.04
2016	17.78	17.41	18.14
2017	18.57	18.19	18.94
2018	18.88	18.50	19.26
2019	19.35	18.96	19.73
2020	23.08	22.66	23.50
2021	21.90	21.49	22.31

* Mid-population is considered from the world population prospectus. https://www.un.org/development/desa/pd/sites/www.un.org.development.desa.pd/files/files/documents/2020/Jan/un_2017_world_population_prospects-2017_revision_databooklet.pdf

**Fig. 3:**
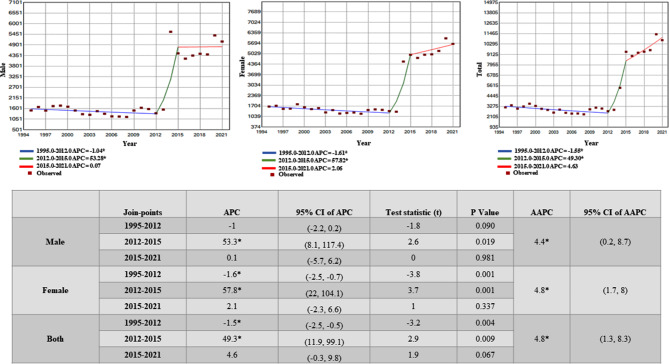
Annual percentage changes (APC) and average annual percentage changes (AAPC) in suicide rates by gender and years using joint point regression analysis

We calculated annual percentage changes (APC) and average annual percentage changes (AAPC) in suicide rates by gender and years using the Joint Point Regression Analysis model (Fig. 3). Here, the positive value of APC suggests an increasing trend, and the negative value of APC suggests a decreasing trend. The analysis of suicide rates over different periods reveals significant insights into trends among males, females, and the total population (Fig. 3).

For males, the APC from 1995 to 2012 was − 1.0% [95% CI: -2.2, 0.2], indicating a decreasing trend, though not statistically significant (t = -1.8, *p* = 0.090). This trend reversed sharply from 2012 to 2015, with an APC of 53.3% [95% CI: 8.1, 117.4], showing a significant increase (t = 2.6, *p* = 0.019). From 2015 to 2021, the trend stabilized with an APC of 0.1% [95% CI: -5.7, 6.2] (t = 0, *p* = 0.981). The average annual percentage change (AAPC) for the entire period was significant at 4.4% [95% CI: 0.2, 8.7].

Among females, the APC from 1995 to 2012 was − 1.6% [95% CI: -2.5, -0.7], reflecting a significant decreasing trend (t = -3.8, *p* = 0.001). However, from 2012 to 2015, there was a significant increase with an APC of 57.8% [95% CI: 22, 104.1] (t = 3.7, *p* = 0.001). The period from 2015 to 2021 showed a non-significant increase with an APC of 2.1% [95% CI: -2.3, 6.6] (t = 1, *p* = 0.337). The AAPC for females over the entire period was significant at 4.8% [95% CI: 1.7, 8].

For both genders combined, the APC from 1995 to 2012 was − 1.5% [95% CI: -2.5, -0.5], indicating a significant decreasing trend (t = -3.2, *p* = 0.004). This was followed by a significant increase from 2012 to 2015 with an APC of 49.3% [95% CI: 11.9, 99.1] (t = 2.9, *p* = 0.009). From 2015 to 2021, the trend showed a non-significant increase with an APC of 4.6% [95% CI: -0.3, 9.8] (t = 1.9, *p* = 0.067). The AAPC for the combined population was significant at 4.8% [95% CI: 1.3, 8.3].

2. Patterns of modes of suicide among the children and adolescents

**Fig. 4:**
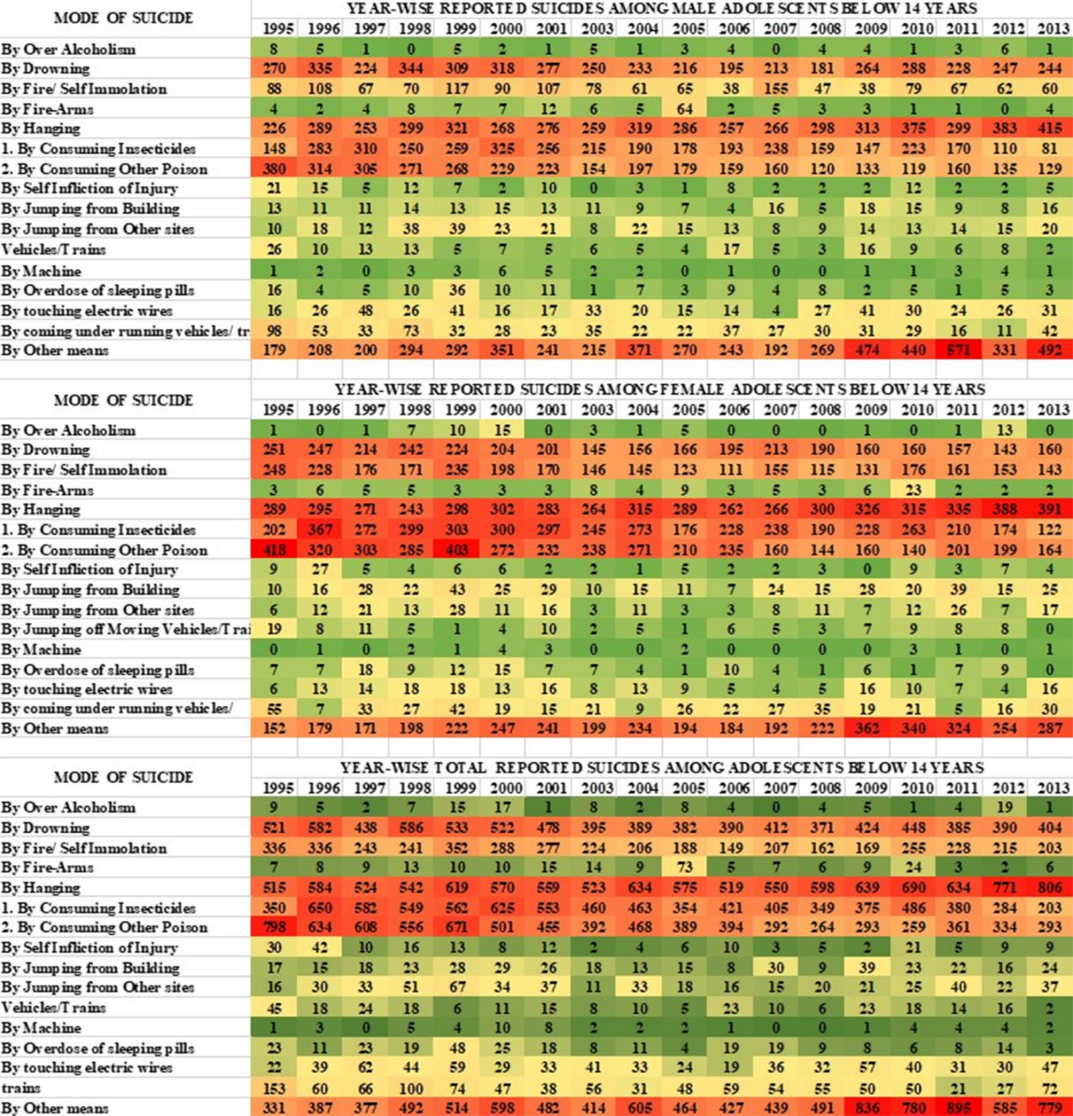
Year-wise reported suicide among children and adolescents (below 14 years) from 1995 to 2013 according to the mode of suicide

NCRB reported the various modes of suicide among children below 14 years of age from the year 1995 to 2013 (Fig. 4). However, data on the modes of suicide was discontinued from 2014 onwards. We analyzed the major modes of suicide among children under 14 years from the available data in the NCRB record. It was observed that hanging was the most common method of suicide among children (< 14 years) in India, accounting for 27.87% of all methods in the year 2013 among both males and females. This trend remained consistent from 1995 to 2013 (Fig. 4). The other prevalent mode of suicide among Indian children was suicide by consuming poison, which accounted for 17.15% of all suicides among children (< 14 years) only in India in 2013 in both genders. These modes of suicide involve ingesting a toxic substance, such as pesticides or drugs. Suicide by drowning was notable in number accounting for 16.41% of all reported cases in 1995, which later decreased to around 3% by 2013. Suicide by fire/self-immolation during 1995 was comparatively high in number, but afterward, it steadily decreased and by 2013, it accounted for 7% of all reported modes. Jumping from a height is another mode of suicide that is prevalent among Indian children, which according to NCRB data, accounted for 2.13% of all reported suicides in 2013. Firearms, touching electric wires, and coming under running vehicles/trains are relatively less prevalent methods (overall accounting for 4.24%) along with jumping from a height (accounting for 2.13%) among Indian children in this age group in 2013.

There was also observed a gender perspective in the rising suicide scenario particularly in terms of adopted modes. With the rise of suicide among both males and females, hanging was the most common mode of suicide among both male and female children. However, poisoning was more prevalent among female children, accounting for 21% of all suicides among them (< 14 years) only in the year 2013, compared to 13.6% among male children. Overall retrospective data from 1995 to 2013 shows that suicide by consuming poison was decreasing in both genders.

3. Cause-wise reported suicide among children and adolescents in India

**Fig. 5:**
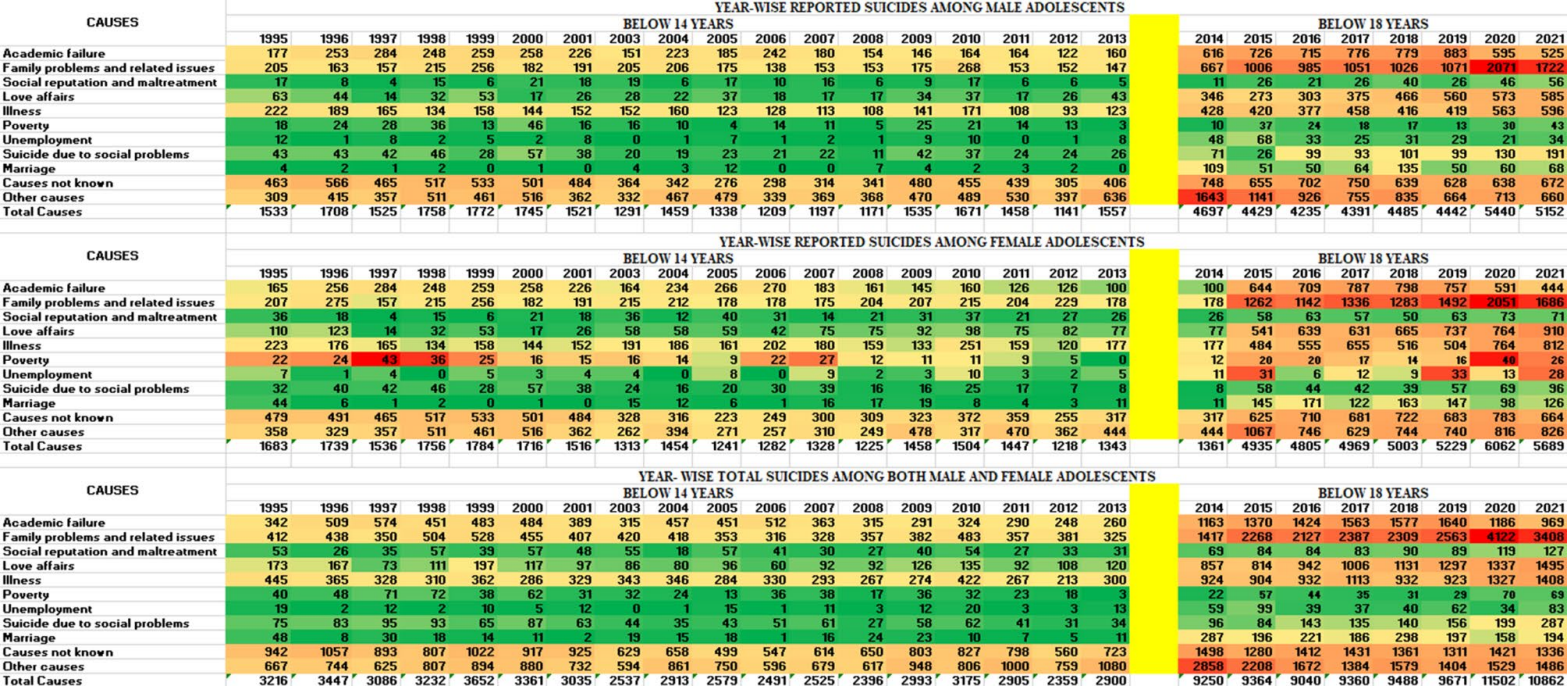
Year-wise reported suicide among children and adolescents according to the causes of suicide from 1995-2021

Figure 5 depicts the heat map describing the distribution of reported suicides among children and adolescents over the years based on various causes. This figure is represented in two parts, the first part covers the period from 1995 to 2013 focusing on children and early adolescents (below 14 years old), while the second part covers the period from 2014 to 2021, focusing on the cause of suicide among children and adolescents (below 18 years old). It can be observed that academic failure, family problems and related issues, love affairs, and illness were the major reasons for suicide among both male and female adolescents in India. Unknown causes and other undefined causes were observed to present a significant section of suicide. As per the trend observed in this analysis, academic failure as a risk of suicide among male adolescents has reduced from 13% during 1995–1999 to 11.1% during 2000–2005 and 9.4% during 2006–2010, while a significant rise was reported with 20.8% during 2011–2015 and 45.5% during 2016–2021. Family problems and related issues have decreased from 7.7% during 1995–1999 to 7.4% during 2000–2005 and 6.8% during 2006–2010 but it has increased to 16.4% during 2011–2015 and 61.4% during 2016–2021. From the year 1995–1999, illness as a causative factor decreased from 13.8 to 11.6% during 2000–2005 and 10.5% during 2006–2010; however, during 2011–2015, the suicide rate due to illness among males rose to 18.7% and this trend during 2016–2021 has significantly increased by 45.1%.

Considering female suicide, academic failure caused cases have decreased from 14.3% during 1995–1999 to 13.5% during 2000–2005 and 10.8% during 2006–2010; however, during 2011–2015 female suicide due to academic failure again increased by 12.9% and this trend during 2016–2021 further raised by 48.2%. Suicides among female adolescents due to family problems and related issues fell from 7.8% during 1995–1999 to 6.9% during 2000–2005 and (6.93%) in 2006–2010. A substantial rise was observed from 2011 to 2015 by 14.5% followed by a 63.7% rise during 2016–2021. The trend of suicide due to illness has experienced a rise of 11.3% from 1995 to 1999 and 11.0% from 2000 to 2005 whereas from 2006 to 2010 the rate has risen to 12.2%, 14.8% from 2011 to 2015, and a significant rise of 50.4% was observed in 2016–2021. In terms of gender, it was shown that from the period 2014 to 2021, suicide due to family problem and academic failure is high among males, while suicide due to love affairs and illness is high among females.

We undertook the cumulative gender-wise difference analysis for the cause of suicide among children and adolescents to understand the differences. We have presented the findings in Table 2. It was observed that there is a significant difference among males and females with respect to various causes of suicide.

**Table 2:** Cumulative gender-wise differences in causes of suicide among children and adolescents (1995–2021)

Cause of death	Male	Female	Chi-square	*P* value
Academic failure	9211	8461	1255.3	< 0.0001
Family problems and related issues	12,893	14,108		
Social reputation and Maltreatment	458	875		
Love affairs	4026	6130		
Illness	6261	7538		
Suicide due to social problems	1376	914		
Marriage	634	1149		
Causes not known	12,981	12,006		
Other causes	15,144	12,720		

### Illness–as a major addressable cause of suicide among children and adolescents

**Fig. 6:**
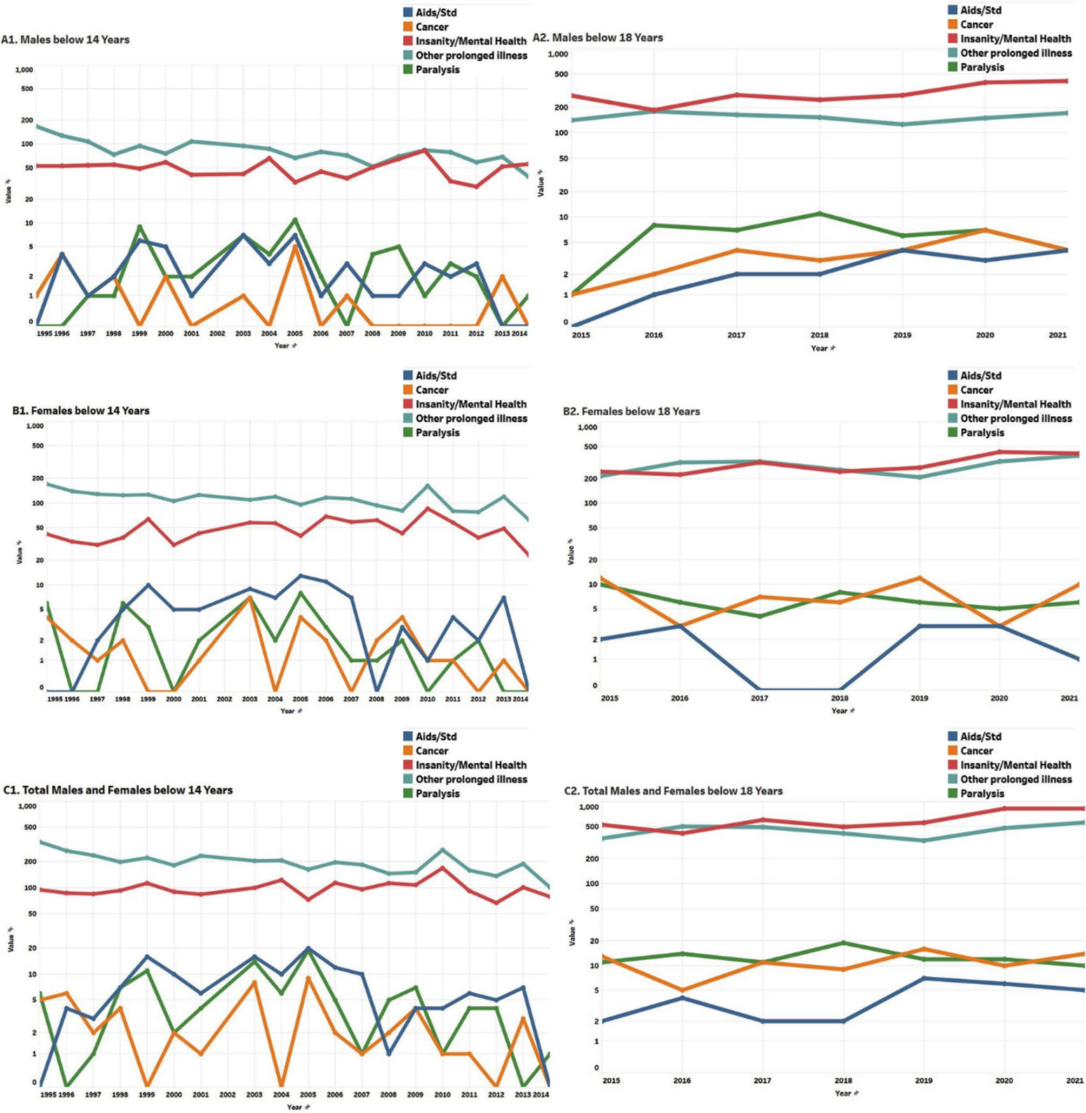
Year-wise reported suicide among children and adolescents based on Illness

In most cases of illnesses leading to suicide, children, and adolescents have been observed taking extreme steps due to unbearable worries without a visible solution and a lack of understanding of the very health conditions. Figure 6 shows that illness is a major cause of suicide among both male and female adolescents. The major illnesses causing suicide, as reported by NCRB, are AIDS/STD, cancer, paralysis, mental illness, and other chronic conditions. Among all the suicides reported due to illness, mental health, and other prolonged illnesses have been consistently high among both males and females as observed from 1995 to 2021 (Fig. 6). Out of the total death due to illness among children and adolescents, death due to mental illness was 7.74% in 1995–1999, but significantly increased after 2011, reaching 47.14% in 2016–2021 (male-59.34%; female-61.33%).

Apart from illness, suicidal deaths due to poverty and unemployment were observable issues among adolescents. Over the period, adolescent suicidal deaths due to poverty (Additional File 1, Figure (A1 & A2) and unemployment (Additional File 1, Figure (B1 and B2)) have consistently evolved as major concerns.

4. Forecasting the future trends of children and adolescent suicide in india for the next 10 years.

**Fig. 7:**
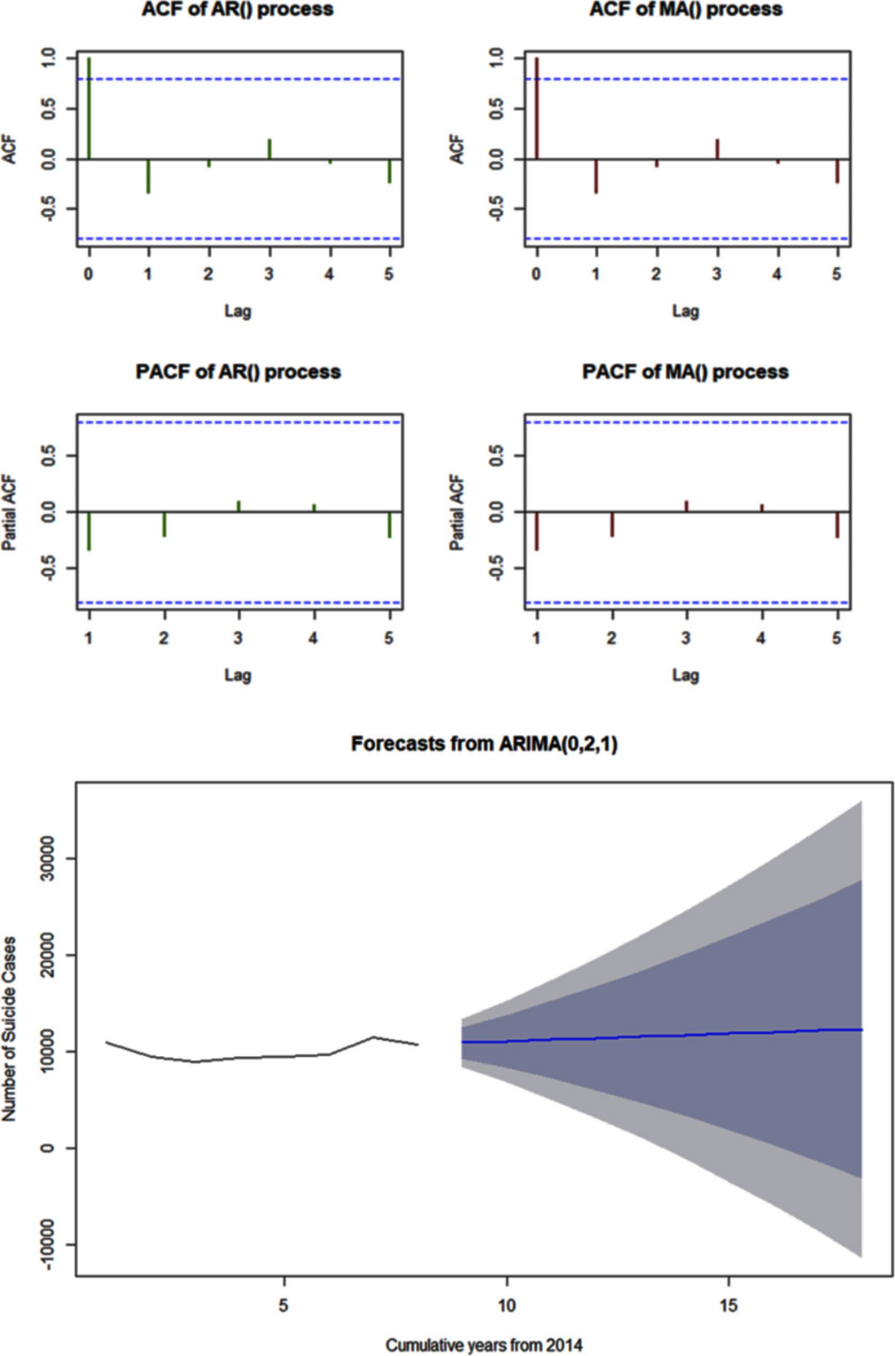
10 year forecasted trend of children and adolescent suicide in India.

We analyzed the time series data from 2014 to the last report available i.e. 2021 to see the future 10-year trend of suicide among children (< 18 years) through forecasting. We adopted the data from 2014 onwards because NCRB started reporting up to 18 years during 2014 and the later period (Fig. 2). We adopted the AREMA model for this time series analysis purpose. As per the finding from the ACF and PACF plots (Fig. 7), the following ARIMA models have been proposed to estimate additional model parameters: ARIMA (0,2,0), ARIMA (0,0,0), ARIMA (0,2,1), ARIMA (1,2,1), ARIMA (2,2,1), ARIMA (1,2,2), ARIMA (0,2,2). The ARIMA (0,2,1) model was found as the best appropriate ARIMA model for Indian children/adolescent suicide rate data because its LL (Log-Likelihood), AIC (Akaike Information Criterion), and BIC (Bayesian Information Criterion) values are the lowest among all suggested models Additional file 3). Additional File 4 shows estimated suicide rates for the next ten years (2022–2031). The graph presenting the forecasted trend of suicide rate for Indian children/adolescents is shown in Fig. 7.

## Discussion

This study explores the pattern, trends, and major risk factors of child and adolescent suicide in India along with forecasting for the next decade. Our study reveals a concerning rise in suicide rates among children and adolescents in India over the past 26 years and the estimate shows an increasing trend through the next decade (Fig. 7). The scenario is particularly concerning among the late adolescents where it shows a stiff rise among females as well as at the overall level (Fig. 3).

The study identifies the means of reported suicide among these populations in India, where hanging was observed as the most common method of suicide among children below 14 years, followed by suicide by consuming poison. These findings highlight the need for stricter regulations and enforcement measures to restrict access to these lethal means, especially among vulnerable populations. Here, it may be mentioned that adolescents from native and indigenous ethnic minority groups are at three times higher risk of suicide than the general population [21]. Globally, similar trends have also been observed in other countries [22, 23]. WHO reported consuming poison as the main method of suicide in an estimated 20% of global suicide cases, most of which are from LMIC countries [24].

The present study reveals an important perspective on gender-wise suicide rates in India in the last 8 years (2014 onwards). While the worldwide suicide trend shows a higher number of suicides occurring among males, in India, this number among female adolescents surpassed males (Fig. 2). This sift is attributed to various causes of suicide, including academic failure, family problems, and illness, emphasizing the complex interplay of social, psychological, and environmental factors contributing to suicidal behavior among both males and females. Suicide due to illness, a major and mostly preventable risk caused a total of 1408 reported suicides in 2021 (NCRB, 2022). Among these illness-associated suicide completers, mental health was observed as a major reason for suicide. It was observed that suicide due to suffering from diseases is more common among females than that of males (Fig. 6) which may have its root in the gender disparity in access to healthcare [25] and well-being in Indian society. This similar pattern is also seen in other countries where cultural attitudes toward mental health contribute to gender differences in healthcare [26–28]. There is a persistent gender paradox globally that, females are more likely to have suicidal thoughts and attempts, while males have higher rates of completed suicide [28, 29].

Similarly, In the context of prolonged illness, adolescents suffer from painful physical distress for a longer period of their lives and lose patience for living; in the absence of adequate resilience skills and social support, they desperately try to escape the pain, causing the adoption of suicide as a major option. Another reason is that due to prolonged illness, expensive medical care expenditures become unaffordable [31, 32] and the financial burden increases the risk of these vulnerable groups slipping into extreme poverty [33, 34].

Furthermore, in the context of the Child Labour (Prohibition and Regulation) Amendment Act 2016 defining the legal age for employment in non-hazardous occupations as 15 years [35], the NCRB report on poverty and unemployment-associated adolescent suicide deaths necessitates the timely and effective implementation of comprehensive social welfare policies and program. Niti Aayog, report 2021 further substantiates this need by indicating the fact that, 7% of India’s population is being pushed into poverty every year due to out-of-pocket expenditures [33], and raising the risks of poor mental health in the country. Similar observations have been reported in a systematic review from South Korea that highlighted low income, unemployment, and financial difficulties as the significant risk factors for suicide [36].

However, there have been several welfare programs initiated under different ministries in the last decade to increase the spending capacity of Indians by making healthcare more affordable for all. “Ayushman Bharat” is a similar umbrella health program at the national level by the Ministry of Health, Government of India while several other programs are available there initiated by various state governments. Though the public subsidy has improved in favor of the poor, it is also a fact that the inequality in the availability and accessibility of healthcare persists which is becoming a major risk factor in mental health care among children and youths in the marginalized section. Additionally, according to the National Mental Health Survey of India 2016, there is a large treatment gap associated with mental health in the country [34]. In the case of adolescents, the lack of proper knowledge and support about mental health creates ambiguity and hampers their scope of going for treatment in everyday life; impaired decision-making increases suicidal tendencies and suicides [36].

In the above context, the national mental health and suicide prevention programs need to explore such barriers and gaps in program implementation towards control and prevention of suicide. National Suicide Prevention Strategy, 2022 has been brought up, primarily aiming for a holistic address of the problem [34]. Developed countries like the United States of America, Canada, and several European countries follow strategies to minimize suicide among adolescents which include different active and passive strategies, such as providing general education about suicide, establishing crisis addressing centers and hotlines, promoting self-esteem and stress management, and developing well-support networks along with regular counseling [37–40]. Such experiences from other countries indicate that the interventions in India require realizing the emerging needs and adopting timely strategies. It is vital to emphasize that there are several major vulnerable communities and sections that are yet to be evaluated on their mental health care and support needs [41].

One major challenge in India is the lack of systematic reporting of mental health problems among children and adolescents. Similarly, there is a major lack of evidence around the risks and rate of suicide among children and adolescents in various settings. For example, a study in Arunachal Pradesh found that there were only two incidents of officially recorded suicide among the Idu Mismi community in a government report out of more than 250 cases collected through investigation during the same time [42]. We understand the low rate of official reporting of suicide has the reasons in follows: (1) it is a taboo/shame for the family to report suicide; (2) Many times, the attributed causes of suicide carry a significant stigma; (3) Suicide is often considered a deviant behavior from community customary perspective in several traditional societies and resolved through traditional councils rather than bringing to the formal system; (4) Suicide deaths/attempts, while reported in local hospitals, are required to be reported as medico-legal cases, which draws a lot of uneasiness. In this scenario, strengthening the reporting process of suicide in vulnerable populations will itself be an advocacy for the community [43].

Among younger children, suicide attempts are often impulsive. It might also be linked to hyperactivity, issues of lack of concentration as well as emotions like depression, confusion, anger, and stress [44]. Adolescents, on the other hand, face higher stigma and feelings of shame after a suicide attempt, which can drive them to repeated attempts [45]. The barriers to accessing mental health services burdened with various regressive cultural and religious beliefs and practices further lead to the rising trend of suicide among adolescents [46]. Bullying is another major public health issue in the context of mental health among adolescents, associated with serious negative outcomes such as depression, anxiety, delinquency, and suicidal thoughts [47, 48]. In India, it has been a major problem in recent times [49].

Social and economic changes, such as rapid urbanization along with increasing inequality, can contribute to feelings of isolation and hopelessness among young people [50]. Changes in family structures, including higher rates of single-parent households and increased parental expectations, may also play a negative role [51]. Academic pressures, fuelled by the competitive nature of the education system, can lead to stress and anxiety, further exacerbating mental health issues [52]. Additionally, the persistent stigma surrounding mental health in India can prevent young people from seeking help [53]. Improving access to mental health services, particularly in rural areas where resources are scarce, is crucial for early intervention and prevention of suicide [34].

This study underscores the need for a comprehensive approach to addressing the complex factors contributing to the rise in suicide rates among children and adolescents in India. By exploring and understanding these factors, policymakers, healthcare professionals, and communities can develop targeted interventions and support systems to prevent suicide and promote mental health among young people.

### Health programs and policies in India around mental health

The Mental Healthcare Bill 2016, which was passed in the Lok Sabha in March 2017 decriminalized suicide and provides mental healthcare and services for persons with mental illness [54]. In recent years, India has initiated and redefined a variety of health programs intended to improve mental health. The National Mental Health Policy (NMHP) of 2014, the Mental Healthcare Act of 2017, Rashtriya Kishore Swasthya Karyakram (RKSK) of 2014, School Health Program (Ayushman Bharat)– 2018, Sarva Shiksha Abhiyan (SSA)– 2011 and National Youth Policy (NYP)-2014, have all been implemented with efforts to enhance the mental health of the children and adolescents of the nation (Table 3). However, as the scenario indicates there is a long way to go to effectively address the obstacles around access and health-seeking behavior around mental health among the children and adolescents in the country [12]. Along with public health supports, social and cultural barriers are to be addressed to ensure the health services be utilized effectively. In the year 2019, India has recorded annual youth suicide rates as 80 per 100,000 in females and 34 per 100,000 in males (compared to 10.4 per 100,000 in the general Indian population) [55]. Though Sect. 309 of the Indian Penal Code (which defines suicide as a criminal act and a non-cognizable offense) has been removed, suicide-related news is still published along with crime news in various Indian newspapers [56]. Similarly, the retrospective data of NCRB India describes a consistent rise in suicide among children and youth in the country (Fig. 7).

**Table 3:** Government programs and policies addressing adolescent mental health issues in India

Sl. No.	Name	Department	Aim
1	School Health Program (Ayushman Bharat) − 2018	Department of School Education, Ministry of Human Resource Development and Ministry of Health and Family Welfare	To raise awareness about age-appropriate health and nutrition content, early disease detection and treatment, and yoga and meditation promotion.
2	Rashtriya Kishore Swasthya Karyakram (RKSK) − 2014	Ministry of Health and Family Welfare	To envision adolescents in India being able to reach their full potential and make decisions about their health and well-being that are well-informed and responsible. Mental health is one of the six components of the program.
3	Sarva Sikhya Abhiyan (SSA)– 2011	Ministry of Human Resource Development	To achieve universal access to education and student retention; to close gender and social status inequalities in education; and to improve children’s learning levels.
4	Mental Healthcare Act 2017	National legislation on Mental Healthcare	To ensure that people’s rights are protected from mental disorder to wellness as well as social services and a way of life in a dignified manner, and to ensure that the legislation is in accordance with the United Nations Convention on the Persons with Disabilities’ Rights. (United Nations Convention on Civil and Political Rights)
5	National Mental Health Policy (NMHP)- 2014	Ministry of Health and Family Welfare	To reduce the amount of services disparities, disease burden, and the degree of the impairment as a result of a mental disorder, take into account the sociocultural background of India promoting evidence-based and efficient delivery of services.
6	National Youth Policy (NYP)-2014	Ministry of Youth Affair and Sports	To encourage young people to succeed by achieving their full potential and wellbeing. And make it possible for India to be an appropriate place in the world.

### Way forward

Addressing the rising rate of suicide among children and adolescents in India requires a comprehensive and multifaceted approach. A focused strategy on illness-related suicide is essential, involving healthcare providers, public helplines, psychologists, teachers, and parents practicing empathy and offering timely support to at-risk children and adolescents. Training in life skills, resilience, optimism, and social support should be integrated into daily routines to help individuals cope with stressors effectively [57–59]. Regular health screening, particularly in vulnerable and underprivileged populations, is crucial for the early detection and treatment of health issues that could lead to suicidal behavior. Free counseling services at school and community levels, along with strengthening tele-counseling facilities, including a crisis hotline should be readily accessible to all children and adolescents during their needs [60]. Ensuring gender equity in healthcare access is vital to address disparities and improve mental health outcomes for both males and females. Strengthening the Rashtriya Kishor Swasthya Karyakram (RKSK) like programs through regular evaluation can enhance their effectiveness towards mental health care. Additionally, targeted interventions to reach out to school dropouts are necessary to provide support and prevent associated mental health risks. Implementing strict anti-bullying policies and timely interventions can create a safer environment in schools and colleges. Actively incorporating life skill development activities like yoga and meditation into school curricula can promote resilience from an early age. Continuous evidence generation and third-party evaluations of government programs are essential for refining strategies and effectively addressing adolescent mental health issues.

## Conclusion

This study gives an in-depth understanding of the historical and recent context of rising suicide rates among Indian children and adolescents over the last 26 years. The findings highlight the urgent need for targeted interventions to address the multifaceted factors contributing to this public health crisis. The prevalence of suicide among this vulnerable population is deeply concerning, especially considering India’s status as home to the largest adolescent population globally. The study highlights the need for strict regulations and enforcement measures to restrict access to lethal means of suicide, such as hanging and poisoning. It also emphasizes the importance of addressing the underlying causes, including academic pressure, family problems, relationship factors, mental health issues, poverty, unemployment, and mainly inadequate support systems which contribute to the growing trends of children/adolescent suicide in India. The study highlights the importance of a holistic approach to suicide prevention. Efforts should focus on raising awareness, decreasing stigma, timely planning, and developing early intervention programs. It was understood that a comprehensive approach is required at government agencies, healthcare providers, educational institutions, policymakers, educators, and the community level to create and implement effective suicide prevention strategies.

### Strengths and limitations of the study

The strength of this study lies in its longitudinal analysis, which spans 26 years from 1995 to 2021, offering a comprehensive examination of trends in child and adolescent suicide rates in India. By utilizing the ARIMA (0,2,1) model, the study also forecasts the suicide rate for the next decade, providing valuable insights into potential future scenarios. This foresight can help policymakers and healthcare professionals prepare for potential challenges and allocate resources effectively. Similarly, by using negative binomial regression, we have been able to highlight the trends of suicide over the period.

However, the study also has limitations. The study relies on data from the National Crime Records Bureau (NCRB), which may have limitations in terms of accuracy and completeness. Additionally, the study focuses on children and adolescents, one from 1995 to 2013 and another from 2014 to 2021, as the NCRB’s age-wise reporting may be insufficient. The study does not explore regional variability in children and adolescent suicide rates within India, which could provide additional insights into the factors contributing to suicide. Furthermore, the lack of contextual evidence for the increase in suicide rates weakens the discussion of trends over time.

Despite these limitations, the study’s strengths, particularly its comprehensive analysis and forecasting, offer important information about suicide among children and adolescents in India.

##  Supplementary material

Supplementary Material 1. Year-wise reported suicide among children/adolescents based on poverty (A1 and A2) and unemployment (B1 and B2).

Supplementary Material 2. Population of children and adolescents by age and sex as of 1st March: (1996-2021) (in ‘000)

Supplementary Material 3. AICs, BIC, and AICc values for suggested ARIMA models for children/adolescent suicide rate in India.

Supplementary Material 4. The forecasted value of children and adolescent suicide rate for the next 10 years based on the ARIMA (0,2,1) model with 80% and 95% confidence intervals.

## Data Availability

No datasets were generated or analysed during the current study.

## Abbreviations

NCRB: National crime records bure
WHO: World Health Organisation
SDG: Sustainable development goals
ADSI: Accidental deaths and suicides in India
ARIMA: Auto-Regressive integrated moving average
LL: Log-likelihood
AIC: Akaike information criterion
BIC: Bayesian information criterion
CSR: Crude suicide rate
LCI: Lower confidential interval
UCI: Upper confidential interval
APC: Annual percentage changes
AAPC: Average annual percentage changes
NMHP: National mental health policy
NYP: National youth policy

## Declarations

### Patient and public involvement

No patients were involved.

## References

[1] Hughes JL, Horowitz LM, Ackerman JP, Adrian MC, Campo JV, Bridge JA. Suicide in young people: screening, risk assessment, and intervention. BMJ. 2023;381. 10.1136/bmj-2022-070630. 10.1136/bmj-2022-070630 PMC11741005 37094838

[2] Campisi SC, Carducci B, Akseer N, Zasowski C, Szatmari P, Bhutta ZA. Suicidal behaviours among adolescents from 90 countries: a pooled analysis of the global school-based student health survey. BMC Public Health. 2020;20:1–1. 10.1186/s12889-020-09209-z 10.1186/s12889-020-09209-z PMC7416394 32772922

[3] Denton EG. Take a closer look: Suicide in low-and middle-income countries (LMICs)[Blog post]. Youth Suicide Research website. [Internet]. 2022. https://www.youthsuicideresearch.org/blog/take-a-closer-look-suicide-in-low-and-middle-income-countries-lmicsblog/youthresearchorg#:~:text=Each%20year%2C%20approximately%20800%2C000%20people,%2Dincome%20countries%20(LMIC’s).

[4] Swain PK, Tripathy MR, Priyadarshini S, Acharya SK. Forecasting suicide rates in India: an empirical exposition. PLoS ONE. 2021;16(7):e0255078. 10.1371/journal.pone.0255342. 34324554 10.1371/journal.pone.0255342 PMC8321128

[5] UNICEF India. For every Child. Adolescent Health and Development. [Internet]. 2023. https://www.unicef.org/india/topics/adolescent-health-and-development#:~:text=Empowering%20adolescent%20girls%20and%20boys,between%2010%20to%2019%20years

[6] WHO. SUICIDE. World Health Organization website. [Internet]. 2023. https://www.who.int/news-room/fact-sheets/detail/suicide#:~:text=Suicide%20is%20the%20fourth%20leading,common%20methods%20of%20suicide%20globally

[7] Senapati RE, Parida J, Badamali J, Jena S, Patsani P, Panda A, Behera SS, Pradhan A, Singh PK, Mishra BK, Patra PK. The patterns, trends and major risk factors of suicide among Indian adolescents - a scoping review protocol. PLoS ONE. 2022;17(11):e0277422. 10.1371/journal.pone.0277422. 36395115 10.1371/journal.pone.0277422 PMC9671363

[8] Hossain MM, Purohit N. Improving child and adolescent mental health in India: Status, services, policies, and way forward. Indian J Psychiatry. 2019;61(4):415. Doi- 10.4103%2Fpsychiatry.IndianJPsychiatry_217_18. 31391648 10.4103/psychiatry.IndianJPsychiatry_217_18 PMC6657557

[9] Malhotra S, Patra BN. Prevalence of child and adolescent psychiatric disorders in India: a systematic review and meta-analysis. Child Adolesc Psychiatry Mental Health. 2014;8(1):1–9. 10.1186/1753-2000-8-22. 10.1186/1753-2000-8-22 PMC4113132 25071865

[10] Senapati RE, Jena S, Parida J, Panda A, Patra PK, Pati S, Kaur H, Acharya SK. The patterns, trends and major risk factors of suicide among Indian adolescents–a scoping review. BMC Psychiatry. 2024;24(1):35. 10.1186/s12888-023-05447-8. 38195413 10.1186/s12888-023-05447-8 PMC10775453

[11] Jena S, Parida J, Panda A, Behera SS, Pradhan A, Patra PK, Pati S, Kaur H, Acharya SK. Knowledge, practices and influencing factors defining unhealthy food behavior among adolescents in India: a scoping review. Frontiers in Psychology. 2023 8;14:1161319. 10.3389/fpsyg.2023.1161319 10.3389/fpsyg.2023.1161319 PMC10285663 37359888

[12] Behera P, Parida J, Kakade N, Pati S, Acharya SK. Addressing barriers to mental healthcare access for adolescents living in slums: a qualitative multi-stakeholder study in Odisha, India. Child Youth Serv Rev. 2023;145:106810. 10.1016/j.childyouth.2023;106810.

[13] Meghrajani VR, Marathe M, Sharma R, Potdukhe A, Wanjari MB, Taksande AB, Meghrajani VR Jr, Wanjari M. A Comprehensive Analysis of Mental Health Problems in India and the role of Mental asylums. Cureus. 2023;15(7). 10.7759/cureus.42559. 10.7759/cureus.42559 PMC10460242 37637646

[14] Gaiha SM, Taylor Salisbury T, Koschorke M, Raman U, Petticrew M. Stigma associated with mental health problems among young people in India: a systematic review of magnitude, manifestations and recommendations. BMC psychiatry. 2020;20:1–24. 10.1186/s12888-020-02937-x 10.1186/s12888-020-02937-x PMC7667785 33198678

[15] Patra S, editor. Adolescence in India: issues, challenges and possibilities. Springer Nature; 2022.

[16] Accidental Deaths & Suicides in India. National Crime Records Bureau, Ministry of Home Affairs, Govt. of India. National Crime Records Bureau website. [Internet]. 2022. https://www.india.gov.in/official-website-national-crime-records-bureau

[17] UNICEF. Adolescent Data Portal. Using data for better understand the lives of adolescents: Population and society. [Internet]. 2023. Available From https://data.unicef.org/adp/downloads/#

[18] UNICEF. Adolescent Data Portal. Using data for better understand the lives of adolescents: health and nutrition; Suicide mortality rate. [Internet]. 2019. Available From: https://data.unicef.org/adp/downloads/#

[19] Surveillance Research Program, National Cancer Institute, USA). [Internet]. 2024. https://surveillance.cancer.gov/

[20] Poduri PGS. Time series analysis of Indian suicides: correlation with human development index (HDI). Acta Med Int. 2015;2(1):122. 10.5530/ami.2015.1.21.

[21] Leckning B, Condon JR, Das SK, He V, Hirvonen T, Guthridge S. Mental health-related hospitalisations associated with patterns of child protection and youth justice involvement during adolescence: a retrospective cohort study using linked administrative data from the Northern Territory of Australia. Child Youth Serv Rev. 2023;145:106771. 10.1016/j.childyouth.2022.106771.

[22] Joseph VA, Martínez-Alés G, Olfson M, Shaman J, Gould MS, Keyes KM. Temporal trends in suicide methods among adolescents in the US. JAMA Netw open. 2022;5(10):e2236049. 10.1001/jamanetworkopen.2022.36049. 36223121 10.1001/jamanetworkopen.2022.36049 PMC9557855

[23] Staniszewska A, Lasota D, Kielan A, Brytek-Matera A. Suicide attempts and suicides as a result of poisoning and under the influence of xenobiotics in Poland in 1999–2020. Int J Environ Res Public Health. 2022;19(4):2343. 10.3390/ijerph19042343. 35206532 10.3390/ijerph19042343 PMC8872402

[24] WHO, Suicide. [Internet]. 2024. https://www.who.int/news-room/fact-sheets/detail/suicide

[25] Kshatriya GK, Acharya SK. Gender disparities in the prevalence of undernutrition and the higher risk among the young women of Indian tribes. PloS one. 2016;11(7):e0158308. 10.1371/journal.pone.0158308 10.1371/journal.pone.0158308 PMC4933394 27379521

[26] Gilmour L, Ring N, Maxwell M. The views and experiences of suicidal children and young people of mental health support services: a meta-ethnography. Child Adolesc Mental Health. 2019;24(3):217–29. 10.1111/camh.12328. 10.1111/camh.12328 32677214

[27] Saikia AM, Das J, Barman P, Bharali MD. Internet addiction and its relationships with depression, anxiety, and stress in urban adolescents of Kamrup District, Assam. J Family Community Med. 2019;26(2):108–12. 10.4103/jfcm.JFCM_93_18. 31143082 10.4103/jfcm.JFCM_93_18 PMC6515762

[28] Azad HA, Monuteaux MC, Rees CA, Siegel M, Mannix R, Lee LK, Sheehan KM, Fleegler EW. Child access prevention firearm laws and firearm fatalities among children aged 0 to 14 years, 1991–2016. JAMA Pediatr. 2020;174(5):463–9. 10.1001/jamapediatrics.2019.6227. 32119063 10.1001/jamapediatrics.2019.6227 PMC7052788

[29] Schrijvers DL, Bollen J, Sabbe BG. The gender paradox in suicidal behavior and its impact on the suicidal process. J Affect Disord. 2012;138(1–2):19–26. 10.1016/j.jad.2011.03.050. 21529962 10.1016/j.jad.2011.03.050

[30] Miranda-Mendizabal A, Castellví P, Parés-Badell O, Alayo I, Almenara J, Alonso I, Blasco MJ, Cebria A, Gabilondo A, Gili M, Lagares C. Gender differences in suicidal behavior in adolescents and young adults: systematic review and meta-analysis of longitudinal studies. Int J Public Health. 2019;64:265–83. 10.1007/s00038-018-1196-1. 30635683 10.1007/s00038-018-1196-1 PMC6439147

[31] Woo JA, Maytal G, Stern TA. Clinical challenges to the delivery of end-of-life care. Prim Care Companion J Clin Psychiatry. 2006;8(6):367. Doi- 10.4088%2Fpcc.v08n0608. 17245459 10.4088/pcc.v08n0608 PMC1764519

[32] Patsani P, Parida J, Jena S, Panda A, Patra PK, Pati S, Kaur H, Acharya SK. A scoping review on knowledge, beliefs and practices towards HIV/AIDS among Indian adolescents. Children and Youth Services Review. 2024:107608. 10.1016/j.childyouth.2024.107608

[33] World inquiry report 2022. Paris: World Inequality Lab. [Internet]. 2022. Available From:: https://wir2022.wid.world/www-site/uploads/2023/03/D_FINAL_WIL_COUNTRY_SHEETS_2303.pdf

[34] National Mental Health Survey. 2015–2016. Mental health Systems. Ministry of health and family welfare government of India. [Internet]. 2022. https://main.mohfw.gov.in/sites/default/files/National%20Mental%20Health%20Survey%2C%202015-16%20-%20Mental%20Health%20Systems_0.pdf

[35] The Child Labour (Prohibition and Regulation) Amendment Act. 2016. Ministry of Labour and Employment. Government of India. [Internet].2024. Available From: https://labour.gov.in/whatsnew/child-labour-prohibition-and-regulation-amendment-act-2016

[36] Raschke N, Mohsenpour A, Aschentrup L, Fischer F, Wrona KJ. Socioeconomic factors associated with suicidal behaviors in South Korea: systematic review on the current state of evidence. BMC Public Health. 2022;22(1):129. 10.1186/s12889-022-12498-1. 35042490 10.1186/s12889-022-12498-1 PMC8765829

[37] Wainberg ML, Scorza P, Shultz JM, Helpman L, Mootz JJ, Johnson KA, Neria Y, Bradford JE, Oquendo MA, Arbuckle MR. Challenges and opportunities in Global Mental Health: a research-to-practice perspective. Curr Psychiatry Rep. 2017;19(5):28. Doi: 10.1007%2Fs11920-017-0780-z. 28425023 10.1007/s11920-017-0780-z PMC5553319

[38] Singh OP. National suicide prevention strategy of India: great leap forward in field of mental health. Indian J Psychiatry. 2023;65(1):1–2. 10.4103/indianjpsychiatry.indianjpsychiatry_835_22. 36874511 10.4103/indianjpsychiatry.indianjpsychiatry_835_22 PMC9983457

[39] Centers for Disease Control and Prevention (CDC). Youth suicide prevention programs: a resource guide [Internet]. MMWR Recomm Rep. 1992;41(RR-12):1–12 [Internet]. 2023. https://www.cdc.gov/mmwr/preview/mmwrhtml/00031525.htm

[40] Rickwood D, Paraskakis M, Quin D, Hobbs N, Ryall V, Trethowan J, McGorry P. Australia’s innovation in youth mental health care: The headspace centre model. Early intervention in psychiatry. 2019;13(1):159– 66. 10.1111/eip.12740 10.1111/eip.12740 PMC6585724 30311423

[41] Kshatriya GK, Acharya SK. Gender disparities in the prevalence of undernutrition and the higher risk among the young women of Indian tribes. PLoS ONE. 2016;5(7):e0158308. 10.1371/journal.pone.0158308. 10.1371/journal.pone.0158308 PMC4933394 27379521

[42] Mane T. Underestimation of suicide: a study of the Idu Mishmi tribe of Arunachal Pradesh. Econ Polit Wkly. 2013;48(52):129–33.

[43] Acharya SK, Kshatriya GK. Social Transformation, Identity of Indian Tribes in Recent Time: An Anthropological prospective. Afro Asian Journal of Anthropology and Social Policy. 2014;5(2):73–88. 10.5958/2229-4414.2014.00008.8

[44] Friedman RA. Uncovering an epidemic—screening for mental illness in teens. N Engl J Med. 2006;28(26):2717–9. 10.1056/nejmp068262. 10.1056/NEJMp068262 17192534

[45] Carpiniello B, Pinna F. The reciprocal relationship between suicidality and stigma. Front Psychiatry. 2017;35. 10.3389/fpsyt.2017.00035. 10.3389/fpsyt.2017.00035 PMC5340774 28337154

[46] Rajkumar E, Julia G, Sri Lakshmi K. Prevalence of mental health problems among rural adolescents in India: a systematic review and meta-analysis. Sci Rep. 2022;12:16573. 10.1038/s41598-022-19731-2. 36195719 10.1038/s41598-022-19731-2 PMC9532445

[47] Srabstein JC, Leventhal BL. Prevention of bullying-related morbidity and mortality: a call for public health policies. Bull World Health Organ. 2010;88:403. 20539848 10.2471/BLT.10.077123 PMC2878162

[48] Modi AA. Suicide:a study on the Impact of Social Media on suicide ideation amongst adolescents. New Delhi: Sri Venkateswara College; 2012.

[49] Thakkar N, van Geel M, Vedder PA. Systematic Review of Bullying and Victimization Among Adolescents in India. Int Journal of Bullying Prevention. 2021; 3, 253–269. 10.1007/s42380-020-00081-4

[50] Haque I, Das DN, Patel PP. Spatial segregation in Indian cities: does the city size matter? Environment and Urbanization Asia. 2018; 9(1):52–68. 10.1177/0975425317749657

[51] Vijayakumar L, Daly C, Arafat Y, Arensman E. Suicide prevention in the Southeast Asia region. Crisis: J Crisis Intervention Suicide Prev. 2020;41(Suppl 1):S21–9. 10.1027/0227-5910/a000666. 10.1027/0227-5910/a000666 32208757

[52] Deb S, Strodl E, Sun H. Academic stress, parental pressure, anxiety and mental health among Indian high school students. International Journal of Psychology and Behavioral Science. 2015;5(1):26–34. 10.5923/j.ijpbs.20150501.04

[53] Raghavan R, Brown B, Horne F, Kumar S, Parameswaran U, Ali AB, Raghu A, Wilson A, Svirydzenka N, Venkateswaran C, Kumar M. Stigma and mental health problems in an Indian context. Perceptions of people with mental disorders in urban, rural, and tribal areas of Kerala. International Journal of Social Psychiatry. 2023;69(2):362-9. 10.1177/00207640221091187 10.1177/00207640221091187 PMC9983047 35549575

[54] 309 Bill Crime of Attempt. [Internet]. 2022. Available From: https://en.wikipedia.org/wiki/Section_309_of_the_Indian_Penal_Code

[55] Gupta S, Basera D. Youth suicide in India: a critical review and implication for the national suicide prevention policy. OMEGA-J Death Dying 2021 Doi- 10.1177/00302228211045169 10.1177/00302228211045169 34505537

[56] Menon V, Mani AM, Kurian N, Sahadevan S, Sreekumar S, Venu S, Kar SK, Arafat SY. Newspaper reporting of suicide news in a high suicide burden state in India: is it compliant with international reporting guidelines? Asian J Psychiatry. 2021;60:102647. 10.1016/j.ajp.2021.102647. 10.1016/j.ajp.2021.102647 33887673

[57] Calear AL, Christensen H, Freeman A, Fenton K, Busby Grant J, Van Spijker B, Donker T. A systematic review of psychosocial suicide prevention interventions for youth. European child & adolescent psychiatry. 2016;25:467– 82. 10.1007/s00787-015-0783-4 10.1007/s00787-015-0783-4 26472117

[58] Jena Samanta L, Parida J, Badamali J, Pradhan A, Singh PK, Mishra BK, Patra PK, Pati S, Kaur H, Acharya SK. The incidence, prevalence, and contributing factors of overweight and obesity among adolescent population of India: A scoping review protocol. Plos one. 2022;17(9):e0275172. 10.1371/journal.pone.0275172 10.1371/journal.pone.0275172 PMC9512208 36156092

[59] Rai RK, Kumar C, Singh L, Singh PK, Acharya SK, Singh S. Rising burden of overweight and obesity among Indian adults: empirical insights for public health preparedness. Journal of Biosocial Science. 2021;53(5):709– 23. 10.1017/S0021932020000486 10.1017/S0021932020000486 32962795

[60] Bilsen J. Suicide and youth: risk factors. Frontiers in psychiatry. 2018;9:407738. 10.3389/fpsyt.2018.00540 10.3389/fpsyt.2018.00540 PMC6218408 30425663

