# Supplementary material for: Trajectory of suicide among Indian children and adolescents: a pooled analysis of national data from 1995 to 2021
Source: Child Adolesc Psychiatry Ment Health. 2024 Sep 30;18:123. doi: 10.1186/s13034-024-00818-9 (PMC11443910; doi:10.1186/s13034-024-00818-9)

**Figure Title:** Year-wise reported suicide among children/adolescents based on poverty (A1 and A2) and unemployment (B1 and B2).


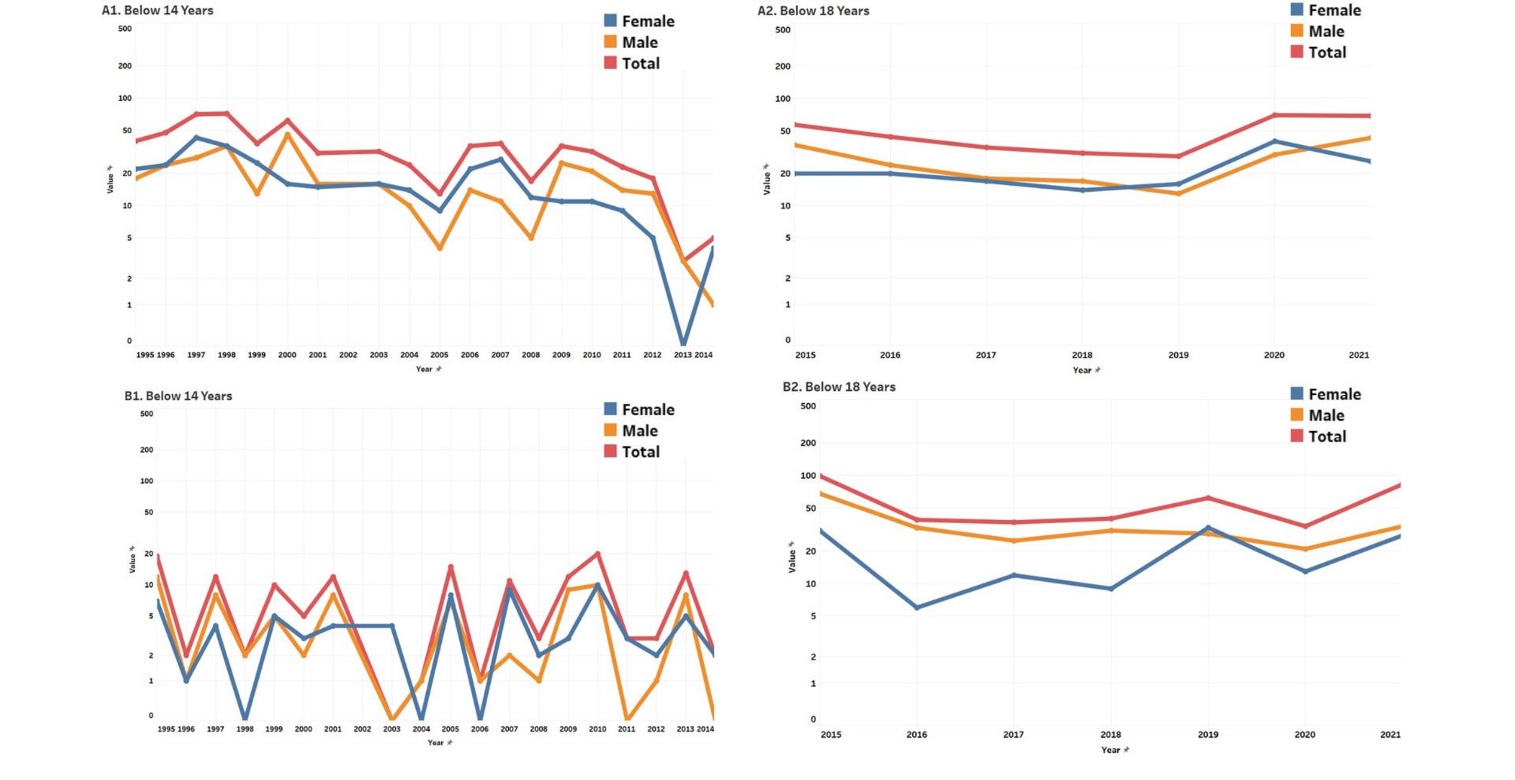

Supplement: Supplementary file 1 — Supplementary Material 1. Year-wise reported suicide among children/adolescents based on poverty (A1 and A2) and unemployment (B1 and B2). [file 13034_2024_818_MOESM1_ESM.docx]
