# Supplementary material for: Trajectory of suicide among Indian children and adolescents: a pooled analysis of national data from 1995 to 2021
Source: Child Adolesc Psychiatry Ment Health. 2024 Sep 30;18:123. doi: 10.1186/s13034-024-00818-9 (PMC11443910; doi:10.1186/s13034-024-00818-9)
Supplement: Supplementary file 2 — Supplementary Material 2. Population of children and adolescents by age and sex as of 1st March: (1996-2021) (in ‘000) [file 13034_2024_818_MOESM2_ESM.docx]

**Table I: Population of children and adolescents by Age and Sex as of 1^st^ March: 1996-2021 (in '000)**

| **Year** | **Male Population** | **Female Population** | **Total population of Children & Adolescents**  **(in '000)** | **Total Suicide in Children & Adolescent Age Group** | **Overall Total population of India (in '000)** |
| --- | --- | --- | --- | --- | --- |
| **Population of Children and Adolescents**  **(0-14 Years) during the period (in '000)** | | | | |  |
| **1996** | 182940 | 169838 | 352778 | 3404 | 934218 |
| **1997** | 182978 | 170666 | 353644 | 3019 | 949878 |
| **1998** | 182564 | 171033 | 353597 | 3262 | 965607 |
| **1999** | 181686 | 170904 | 352590 | 3575 | 981324 |
| **2000** | 180340 | 170243 | 350583 | 3324 | 996944 |
| **2001** | 178518 | 169026 | 347544 | 3007 | 1012386 |
| **2002** | 177148 | 167968 | 345116 | 2880 | 1027607 |
| **2003** | 175645 | 166754 | 342399 | 2576 | 1043534 |
| **2004** | 174106 | 165474 | 339580 | 2913 | 1060024 |
| **2005** | 172689 | 164288 | 336977 | 2555 | 1076934 |
| **2006** | 171483 | 163238 | 334721 | 2464 | 1094126 |
| **2007** | 171494 | 163308 | 334802 | 2479 | 1111446 |
| **2008** | 171569 | 163253 | 334822 | 2381 | 1128571 |
| **2009** | 171735 | 163193 | 334928 | 2951 | 1145513 |
| **2010** | 172006 | 163200 | 335206 | 3130 | 1162283 |
| **2011** | 172386 | 163318 | 335704 | 3035 | 1178889 |
| **2012** | 173589 | 164339 | 337928 | 2738 | 1195035 |
| **2013** | 175081 | 165706 | 340787 | 2891 | 1211575 |
| **Population of Children and Adolescents**  **(0-19 Years) during the period (in '000)** | | | | |  |
| **2014** | 231782 | 219913 | 451695 | 5369 | 1228506 |
| **2015** | 232637 | 220533 | 453170 | 9424 | 1245827 |
| **2016** | 256736 | 233560 | 490296 | 8951 | 1291074 |
| **2021** | 248557 | 225715 | 474272 | 10730 | 1363006 |

**Source:** The Register General of India. Population Projections for Indian States. Ministry of Home Affairs, Govt of India, New Delhi. Available at: <https://censusindia.gov.in/nada/index.php/catalog/32987/download/36805/48978_1991_POP.pdf>

**Table II:** AICs, BIC, and AICc values for suggested ARIMA models for children/adolescent suicide rate in India.

| Model | LL | AIC | BIC |
| --- | --- | --- | --- |
| ARIMA (0,2,0) | -45.11 | 92.22 | 91.82 |
| ARIMA (0,0,0) | -51.6 | 107.19 | 106.77 |
| ARIMA (0,2,1) | -37.07 | 78.14 | 76.91 |
| ARIMA (1,2,1) | -36.93 | 79.86 | 78.01 |
| ARIMA (2,2,1) | -36.59 | 81.18 | 78.72 |
| ARIMA (1,2,2) | -36.65 | 81.29 | 78.83 |
| ARIMA (0,2,2) | -36.73 | 79.45 | 77.60 |

**Table III:** The forecasted value of children and adolescent suicide rate for the next 10 years based on the ARIMA (0,2,1) model with 80% and 95% confidence intervals.

| **Year** | **Forecast Value** | **Lo 80** | **Hi 80** | **Lo 95** | **Hi 95** |
| --- | --- | --- | --- | --- | --- |
| **2022** | 10886.58 | 9280.512 | 12492.65 | 8430.31 | 13342.86 |
| **2023** | 11043.17 | 8275.26 | 13811.07 | 6810.018 | 15276.32 |
| **2024** | 11199.75 | 7189.161 | 15210.34 | 5066.084 | 17333.42 |
| **2025** | 11356.33 | 6000.095 | 16712.57 | 3164.674 | 19547.99 |
| **2026** | 11512.92 | 4707.478 | 18318.36 | 1104.897 | 21920.94 |
| **2027** | 11669.5 | 3314.967 | 20024.03 | -1107.66 | 24446.66 |
| **2028** | 11826.08 | 1827.021 | 21825.15 | -3466.16 | 27118.33 |
| **2029** | 11982.67 | 248.0007 | 23717.33 | -5963.96 | 29929.29 |
| **2030** | 12139.25 | -1418.07 | 25696.57 | -8594.88 | 32873.38 |
| **2031** | 12295.83 | -3167.55 | 27759.21 | -11353.4 | 35945.03 |
