# Supplementary material for: Trajectory of suicide among Indian children and adolescents: a pooled analysis of national data from 1995 to 2021
Source: Child Adolesc Psychiatry Ment Health. 2024 Sep 30;18:123. doi: 10.1186/s13034-024-00818-9 (PMC11443910; doi:10.1186/s13034-024-00818-9)
Supplement: Supplementary file 3 — Supplementary Material 3. AICs, BIC, and AICc values for suggested ARIMA models for children/adolescent suicide rate in India. [file 13034_2024_818_MOESM3_ESM.docx]

**Table:** LL, AIC, and BIC values for suggested ARIMA models for children and adolescent suicide rate in India.

| **Model** | **LL** | **AIC** | **BIC** |
| --- | --- | --- | --- |
| **ARIMA (0,2,0)** | -45.11 | 92.22 | 91.82 |
| **ARIMA (0,0,0)** | -51.6 | 107.19 | 106.77 |
| **ARIMA (0,2,1)** | -37.07 | 78.14 | 76.91 |
| **ARIMA (1,2,1)** | -36.93 | 79.86 | 78.01 |
| **ARIMA (2,2,1)** | -36.59 | 81.18 | 78.72 |
| **ARIMA (1,2,2)** | -36.65 | 81.29 | 78.83 |
| **ARIMA (0,2,2)** | -36.73 | 79.45 | 77.60 |
