# Supplementary material for: Trajectory of suicide among Indian children and adolescents: a pooled analysis of national data from 1995 to 2021
Source: Child Adolesc Psychiatry Ment Health. 2024 Sep 30;18:123. doi: 10.1186/s13034-024-00818-9 (PMC11443910; doi:10.1186/s13034-024-00818-9)
Supplement: Supplementary file 4 — Supplementary Material 4. The forecasted value of children and adolescent suicide rate for the next 10 years based on the ARIMA (0,2,1) model with 80% and 95% confidence intervals. [file 13034_2024_818_MOESM4_ESM.docx]

**Table 2:** The forecasted value of children/adolescent suicide rate for the next 10 years based on the ARIMA (0,2,1) model with 80% and 95% confidence intervals.

| **Year** | **Forecast Value** | **Lo 80** | **Hi 80** | **Lo 95** | **Hi 95** |
| --- | --- | --- | --- | --- | --- |
| **2022** | 10886.58 | 9280.512 | 12492.65 | 8430.31 | 13342.86 |
| **2023** | 11043.17 | 8275.26 | 13811.07 | 6810.018 | 15276.32 |
| **2024** | 11199.75 | 7189.161 | 15210.34 | 5066.084 | 17333.42 |
| **2025** | 11356.33 | 6000.095 | 16712.57 | 3164.674 | 19547.99 |
| **2026** | 11512.92 | 4707.478 | 18318.36 | 1104.897 | 21920.94 |
| **2027** | 11669.5 | 3314.967 | 20024.03 | -1107.66 | 24446.66 |
| **2028** | 11826.08 | 1827.021 | 21825.15 | -3466.16 | 27118.33 |
| **2029** | 11982.67 | 248.0007 | 23717.33 | -5963.96 | 29929.29 |
| **2030** | 12139.25 | -1418.07 | 25696.57 | -8594.88 | 32873.38 |
| **2031** | 12295.83 | -3167.55 | 27759.21 | -11353.4 | 35945.03 |
